# Genetic Identification of *Echinococcus granulosus* Isolates in Hamadan, Western Iran

**Published:** 2018

**Authors:** Mohammad MATINI, Maliheh ROOSTAEI, Mohammad FALLAH, Amir Hossein MAGHSOOD, Massoud SAIDIJAM, Majid FASIHI HARANDI

**Affiliations:** 1. Dept. of Medical Parasitology and Mycology, School of Medicine, Hamadan University of Medical Sciences, Hamadan, Iran; 2. Dept. of Molecular Medicine and Genetics, School of Medicine, Hamadan University of Medical Sciences, Hamadan, Iran; 3. Dept. of Medical Parasitology, School of Medicine, Kerman University of Medical Sciences, Kerman, Iran

**Keywords:** *Echinococcus granulosus*, Genotype, Iran, ITS1, Humans, Livestock, PCR-RFLP

## Abstract

**Background:**

Cystic echinococcosis is a zoonotic infection and considered as a major economic and public health concern worldwide. This research was conducted to determine genotypic characteristics of livestock and human hydatid cyst isolates from Hamadan area, western Iran.

**Methods:**

Sampling was conducted in Hamadan industrial slaughterhouse and Beast Hospital of Hamadan City, western Iran, from 2015 to 2016. Overall, 74 livestock isolates including 69 sheep, 3 cattle and 2 goats and 9 human hydatid cysts were genotyped by PCR amplification of the rDNA ITS1 region and followed restriction fragment length polymorphism (RFLP) analysis with four restriction endonuclease enzymes, *RsaI*, *HpaII*, *AluI*, and *TaqI*, and sequencing.

**Results:**

The PCR amplicon size of each isolate was approximately 1 kb which was the same with that of sheep strain. According to the RFLP patterns, the isolates belonged to a single species, *E. granulosus* sensu stricto (G1–G3 complex). Furthermore, sequencing of representative amplicons confirmed that the RFLP-genotyped isolates corresponded to *E. granulosus* sensu stricto.

**Conclusion:**

*E. granulosus* sensu stricto is the prevailing species of *E. granulosus* sensu lato in the region and pointed out the importance of sheep/dog cycle in human transmission.

## Introduction

Cystic echinococcosis (CE) is a cosmopolitan zoonotic infection caused by the larval stage of a taeniid tape-worm, *Echinococcus granulosus* sensu lato. The parasite life cycle engages two hosts including carnivorous, as a definitive host, and herbivorous, as an intermediate host. In the domestic life cycle, dog and livestock have the main role in survival and distribution of the infection. Hydatid cyst, the encysted larval stages of the parasite, develops from ingested eggs in inner organs of the intermediate host animals and gradually grows (1). Hydatid cyst establishes generally in liver (more than 65% of the cases) and lungs (25% of the cases) in intermediate hosts, however, other organs can be involved, including heart, kidneys, spleen, bone, eyes, muscles and central nervous system (2). A human can be infected by the metacestode stage of the parasite as an accidental intermediate host (1).

CE is considered a public health problem and economic concern in many countries worldwide (3). This zoonotic infection has extensive distribution in the world with highly endemic regions including Australia, China, the Middle East, Western and Central Asia, Northern and Eastern Africa and Southern America (2). Echinococcosis/hydatidosis should be considered as a prevalent infection in Iran with a prevalence rate of 5%–49% in stray dogs and with an average of 6.73% among livestock (4). Phylogenetic analyses and molecular epidemiological studies based on mitochondrial genes (COI, NDI and ATP6) and nuclear rRNA genes (ITS1) revealed that *E. granulosus* s.l. is a set of species/genotypes including *E. granulosus* sensu stricto (G1–G3 complex), *E. equinus* (G4), *E. ortleppi* (G5) and *E. canadensis* (G6–G10) (5–7). Situation of *E. canadensis* genotypes is controversial and it suggested to be divided into two distinct species consist of *E. canadensis* (G8 and G10), cervid genotypes, and *E. intermedius,* (G6/G7), camel/pig genotypes (3, 8).

Variation in species/genotypes of *E. granulosus* s.l. are reflected in morphological and biological characteristics of the parasite and it can influence the life cycle pattern, host specificity, development rates, pathogenicity, treatment, transmission dynamics, epidemiology and finally control of CE (9).

Morphological and molecular epidemiological studies have been conducted in Iran showed existence of *E. granulosus* sensu stricto and camel genotype in livestock, dog and human (10–14). Hamadan Province is one of the endemic regions of echinococcosis/hydatidosis in Iran (15, 16). However, there is little information about the genetic characterization of *E. granulosus* s.l. in this area.

This study was conducted to investigate the species/genotypes of the parasite in the region by using PCR-RFLP and nucleotide sequences analysis of the ITS1-rDNA locus.

## Materials and Methods

### Parasites

During Mar 2015 to Apr 2016, livestock hydatid cysts including sheep, cattle, goats and human hydatid cysts were collected from Hamadan industrial slaughterhouse and Beast Hospital of Hamadan city, western Iran, respectively. Fertile hydatid cysts with colorless transparent fluid and whitish germinal layer were selected for molecular analysis. Aspirated protoscoleces from individual hydatid cyst washed three times with sterile normal saline solution and preserved in 70% (v/v) ethanol at −20 °C, until use for molecular processes.

### DNA extraction

Total genomic DNA (gDNA) extraction was performed on 50 mg protoscolices pellet of each individual hydatid cyst by using High Pure PCR Template Preparation Kit (Roche Diagnostics, Mannheim, Germany) following the manufacturer’s recommended protocol. The extracted gDNA was evaluated by 0.8% agarose gel electrophoresis and spectrophotometer (NanoDrop-ND1000) and the DNA stored at −20 °C until molecular analysis.

### PCR amplification and RFLP analysis

PCR amplification was performed on DNA samples to amplify the ITS1 rDNA region of *E. granulosus* s.l. using forward (EgF: 5′ GTC GTA ACA AGG TTT CCG TAG G 3′) and reverse (EgR: 5′ TAG ATG CGT TCG AAG TGT CG 3′) primers (17). The PCR reaction was done in a final volume of 50 μl, including 7 μl gDNA, 25 pmol of each primer, 5 μl buffer 10X, 2 μl dNTP, 2 μl MgCl2, 2 unit Taq DNA polymerase (Sinagene Company) and 29 μl distilled water. Negative (no added DNA) and positive controls were included in PCR experiment. The PCR conditions were as follows: one cycle of primary denaturation (2 min at 95 °C), followed by 35 cycles of denaturation (30 sec at 94 °C), annealing (45 sec at 55 °C), extension (1 min at 72 °C), and followed by a final extension (5 min at 72 °C). Then, the PCR product was evaluated by electrophoresis in a 1% (w/v) Tris-borate/EDTA (TBE) agarose gels stained with SYBR Safe DNA gel stain (Invitrogen).

RFLP analysis of the PCR product was performed by four restriction enzymes including *RsaI*, *HpaII*, *AluI*, and *TaqI* which identify nucleotide sequences of GT/AC, C/CGG, AG/CT, and T/CGA, respectively. The digestion reaction was carried out on 10 μl of the PCR amplicon in a final 20 μl volume and incubation at 37 °C for 4 h following the manufacturer’s instructions (fermentas, Germany), with the exception of TaqI, incubated at 65 °C. The restriction fragments were conducted to 2% agarose gel electrophoresis and visualized under UV light after SYBR Safe DNA gel staining.

### Sequence analysis

Representative PCR amplicons of different intermediate host including sheep, cattle, goat, and human were subjected to sequencing using Applied Biosystems Automated 3730xl DNA Analyzer (Bioneer Inc., Korea) and the same primers utilized in the primary amplification. Sequence editing and alignment were done by Chromas software, ver. 2.6, and MultAlin program (18). Homology survey of the consensus sequences was carried out by using the BLASTn program (https://blast.ncbi.nlm.nih.gov/Blast.cgi) and comparison with other reference sequences in GenBank.

## Results

**Fig. 1:**
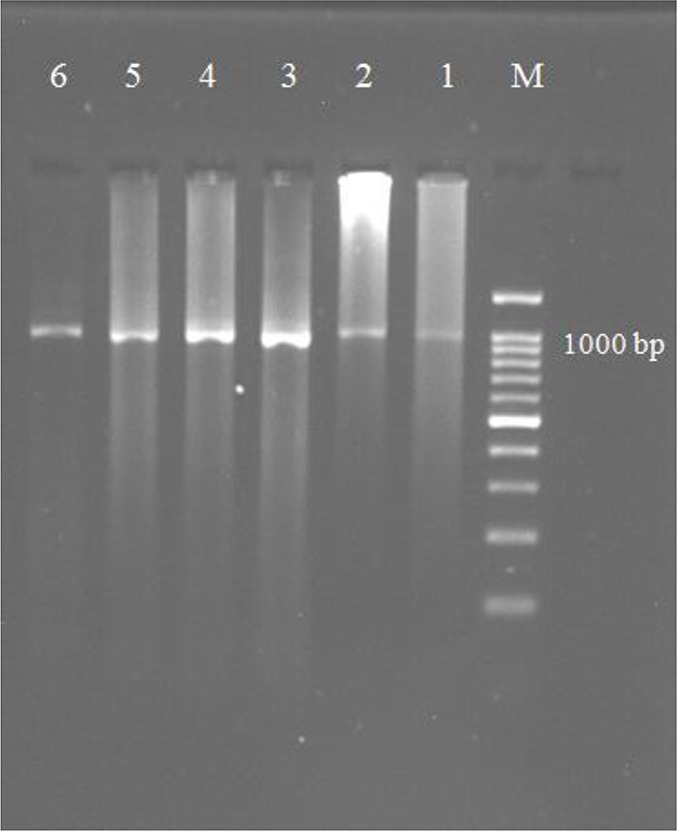
Agarose gel electrophoresis of PCR-ITS1 amplification of the 1000 bp fragment from *E. granulosus* isolates. Lane M: DNA marker (100 bp); Lane 1–6: some of the *E. granulosus* isolates

The PCR amplification of the ITS1 region of rDNA was successfully performed on 74 animal DNA (69 sheep, 3 cattle, and 2 goats) and 9 human DNA samples and produced amplicons of approximately 1 kb in length which are similar to the sheep strain (Fig. 1).

**Fig. 2:**
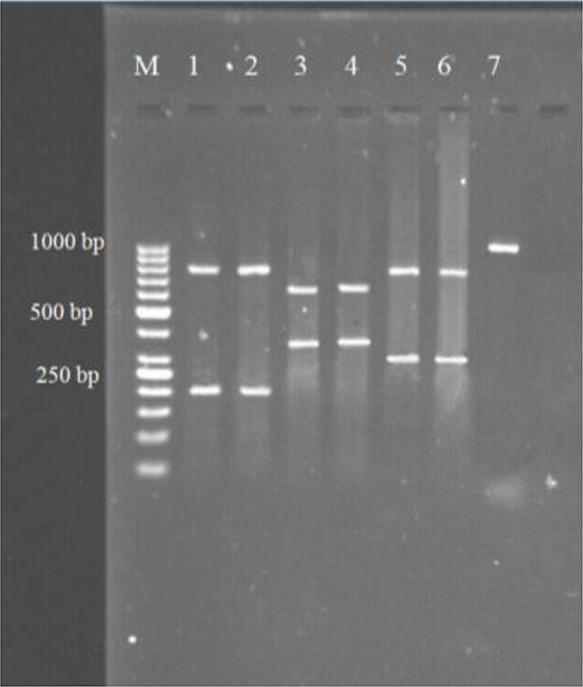
Representative RFLP analysis patterns of the amplified ITS1 rDNA region of *E. granulosus* isolates were digested by *AluI* (lane 1 & 2), *RsaI* (lane 3 & 4), *HpaII* (lane 5 & 6) and *TaqI* (lane 7)

The samples used for PCR tests were 36 liver and 47 lung hydatid cysts. No amplification was observed in negative control in each PCR run. RFLP analysis of the ITS1 rDNA region was done by using the four restriction endonucleases and one identical pattern was obtained from the animal and human hydatid cyst isolates by each of the restriction enzymes. The size of fragments produced by the restriction enzymes was as follows: *AluI*: 200 and 800 bp; *RsaI*: 345 and 655; HpaII: 300 and 700 bp; TaqI: without any digestion (Fig. 2). The four RFLP patterns indicate that the isolates belong to *E. granulosus* s.s. (19).

**Fig. 3:**
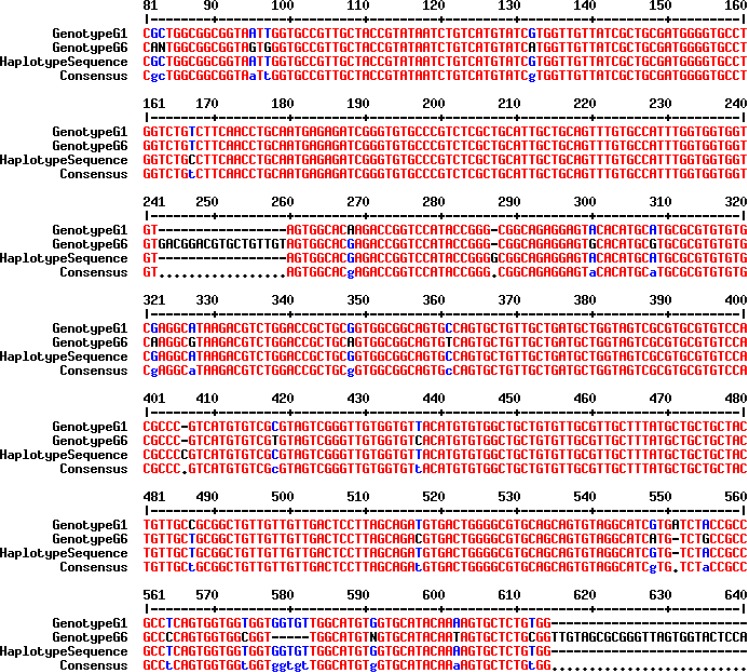
Partial sequence (MF004421) alignment of the ITS1 rDNA region of *E. granulosus* isolates was compared with reference strains (G1 genotype: GenBank Accession No. AJ237777 & G6 genotype: Accession No. AJ237776)

Fourteen representative amplicons of ITS1-PCR, including animal and human isolates, were subjected to sequencing. Partial consensus sequences of *E. granulosus* ITS1 rDNA region of the isolates were achieved and compared with the reference sequence genotype G1 (Accession No. AJ237777) (20) and other sequences deposited in GenBank. Alignment of the obtained sequences with the reference sequence revealed 99% homology and indicated that the isolates corresponded to *E. granulosus* genotype G1 (Fig. 3). Finally, a consensus sequence was deposited at GenBank database under accession numbers MF004421.

## Discussion

As for any infectious disease, control of hydatid disease requires a clear species-level classification and genetic identification of the species. Comprehensive molecular genetic analysis of *E. granulosus*, based on mitochondrial and nuclear rRNA genes, revealed some of the strains of *E. granulosus* can be considered as distinct species (3). PCR–RFLP analysis of ITS1 region of *Echinococcus* can distinguish *E. granulosus* s.s., *E. equinus, E. ortleppi*, G6/G7 genotypes, *E. multilocularis*, *E. vogeli* and *E. oligarthrus* (10, 21). Human can be infected by all *E. granulosus* genotypes except G4 (3, 22). Thus, various animals can play a role in the epidemiology of human echinococcosis/hydatidosis and design an appropriate strategy to control the infection requires a good understanding of transmission cycle in each region.

Until now, two main genotypes including G1–G3 complex and G6 have been reported from Iran based on mitochondrial genes (cox1 and nad1) sequencing and PCR-RFLP analysis of rDNA-ITS1 (10–14, 19, 23). In this study, *E. granulosus* s.s. was detected in the examined livestock and human hydatid cysts and it indicates the genotypic cluster G1–G3 is the dominant genotype of *E. granulosus* in the area. This finding is consistent with the previous studies from other parts of Iran that they have reported *E. granulosus* s.s. as the prevailing type of the parasite in animals and human isolates (10–14, 19). Moreover, some of the workers have reported genotype G1 (common sheep strain) as the only genotype in animal and human hydatid cysts (19, 23).

In the present study, genotype G6 was not detected among examined animal and human isolates which may be due to the absence of camel, the natural intermediate host of the camel strain, in the region. Some species/strains of *E. granulosus*, especially sheep strain (G1) has a higher potential for adaptation to a wide range of host species under various environmental conditions. Thus, this strain is likely the most widespread *E. granulosus* strain worldwide and the common etiologic agent of human CE (22).

Various molecular studies were conducted for genetic identification of *E. granulosus* in the endemic areas in the world. Villalobos and colleagues reported genotypes G1 and G7 in pigs from Mexico, known as a hypoendemic area of echinococcosis, using ITS1-PCRRFLP analysis and mitochondrial genes sequencing (24). In Argentina, a hyperendemic in South America, 76 hydatid cysts from cattle and sheep were genetically identified and *E. granulosus* s.s. genotype G1 was dominant genotype and two isolates from sheep and cattle were detected as genotype G2 and G5, respectively (25). Human CE was investigated in South Africa. In this study, 32 human isolates were subjected to genetic identification using PCR-RFLP and sequencing of 12s rRNA gene. The most of the isolates [26/32] belonged to *E. granulosus* s.s., and G6/G7 and G5 genotypes constituted five and one of the isolates, respectively (26). However, in some areas of Africa such as Mauritania and Sudan, G6 is the prevailing genotype of the parasite in sheep, cattle, camel and human (27, 28). In Russia, an endemic area for both multilocular and unilocular hydatid cyst, genetic identification of *Echinococcus* spp. was conducted. Seventy-five *Echinococcus* isolates obtained from 14 host species were identified using mitochondrial DNA sequencing. In this study, in addition to *E. granulosus* s.s., genotype G6, G8 and G10 and also *E. multilocularis* were detected in the different regions of Russia (29). In another study on human and dog isolates in northwest of China, the Xinjiang Uygur Autonomous Region, one of the important foci of human CE in the world, the most of the human [45/47] and dog [42/45] isolates was detected as G1 genotype and the others were G6 genotype (30). In Turkey, the neighbor of Iran, 112 livestock isolates including sheep [100] and cattle [12] hydatid cysts were genetically categorized based on the mitochondrial cox1 gene. Genotype G1 was found the major genotype [107/112] and five isolates (two sheep and three cattle) corresponded to G3 genotype (31).

In the present study, the results of representative sequencing of the rDNA-ITS1 region of *E. granulosus* isolates were consistent with those of genotyping of the isolates by RFLP analysis and the obtained G1 sequences had high similarity (99%) with Bowles isolate (G1 reference sequence, AJ237777) (20) and other G1 sequences.

## Conclusion

This research as the first genetic characterization of *E. granulosus* in the Hamadan area showed that *E. granulosus* s.s (genotype G1–G3 complex) is the common species/genotypes of *E. granulosus* s.l. in the area and the sheep/dog cycle is the most probable parasite life cycle in this area. Thus, further genetic studies are needed to exactly determine the genotypes of *E. granulosus* s.s in the region. Eventually, this information may be considered when implementing hydatidosis control programs, because antigenic variation and differences in other biological characteristics of *Echinococcus* species were reported (9).
